# Supplementary material for: Acid Ceramidase Inhibitor LCL-805 Antagonizes Akt Signaling and Promotes Iron-Dependent Cell Death in Acute Myeloid Leukemia
Source: Cancers (Basel). 2023 Dec 15;15(24):5866. doi: 10.3390/cancers15245866 (PMC10742122; doi:10.3390/cancers15245866)
Supplement: Supplementary file 1 [file cancers-15-05866-s001.zip › cancers-2702713-supplementary.pdf]

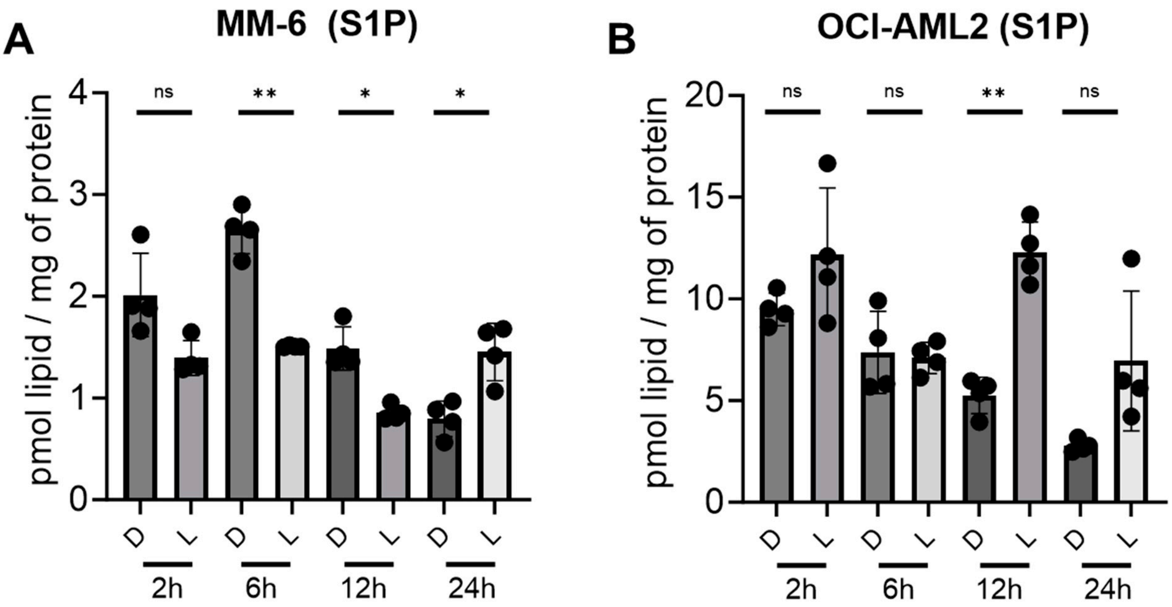

Figure S1. Effect of LCL-805 on sphingosine-1-phosphate levels. Sphingolipid profiling of MM-6 (A) and OCI-AML2 (B) cells treated with vehicle (DMSO) or LCL-805 (15 μM) for the indicated times. Levels of sphingosine-1-phosphate were evaluated by liquid chromatography mass spectrometry. Bars represent the average of four technical replicates from a representative experiment. Error bars represent +/- standard deviation. Statistical analyses represent Welch's ANOVA with Dunnett's T3 multiple comparisons test. * $p < 0.05$, ** $p < 0.005$, ns = non-significant. D = DMSO; L = LCL-805.

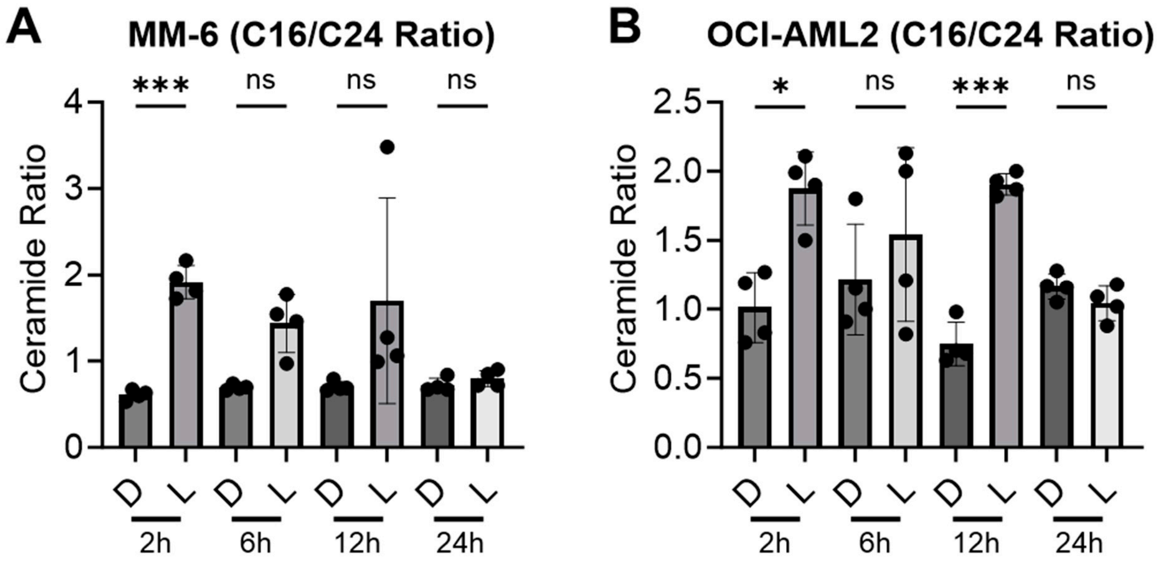

Figure S2. LCL-805 increased the C16-/C24-ceramide ratio. Sphingolipid profiling of MM-6 (A) and OCI-AML2 (B) cells treated with vehicle (DMSO) or LCL-805 (15 μM) for the indicated times. Levels of ceramide were evaluated by liquid chromatography mass spectrometry. Bars represent the average of four technical replicates from a representative experiment. Error bars represent +/- standard deviation. Statistical analyses represent Welch's ANOVA with Dunnett's T3 multiple comparisons test. * $p < 0.05$, *** $p < 0.001$, ns = non-significant. D = DMSO; L = LCL-805. C## = fatty acid chain length.

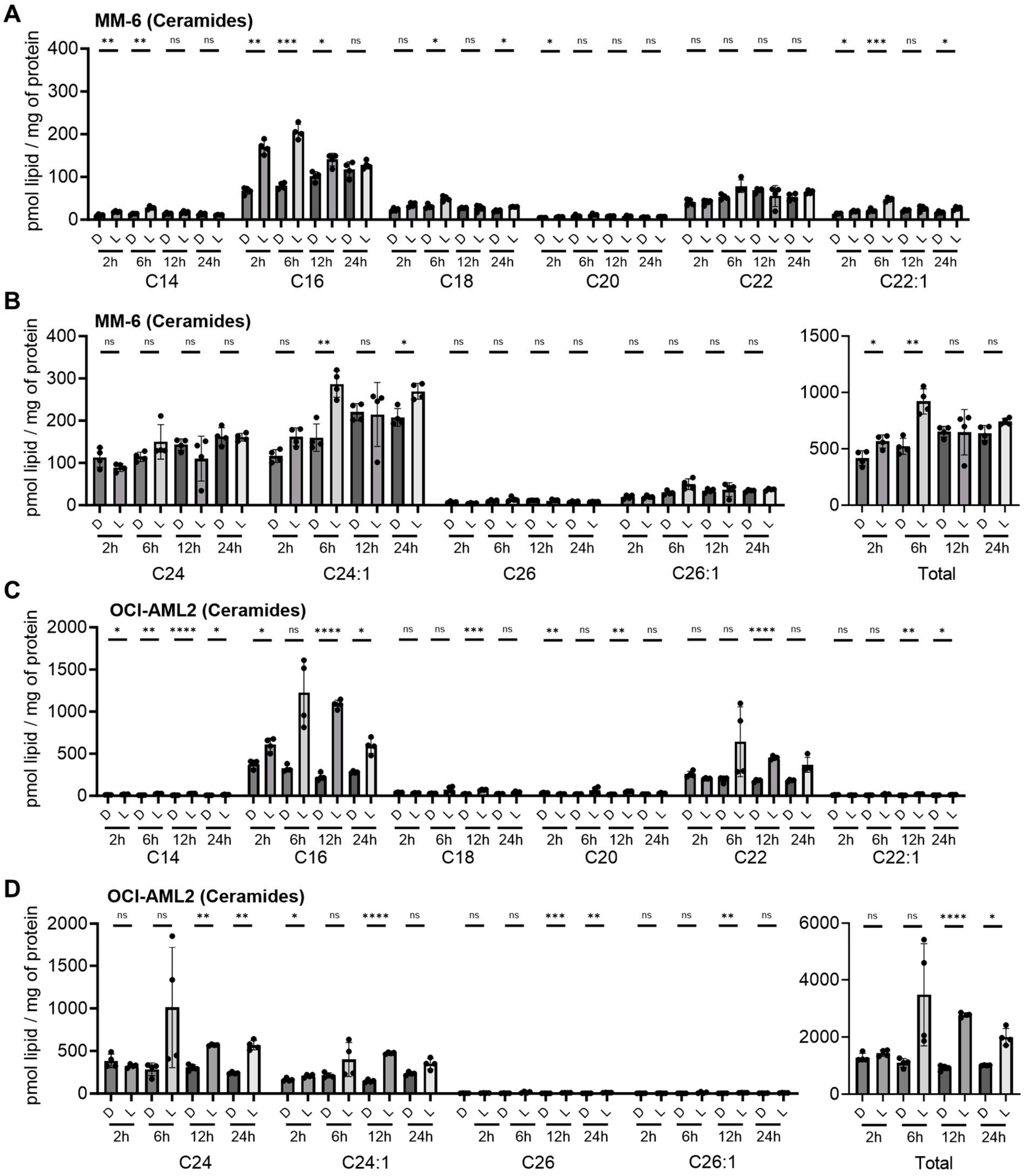

Figure S3. Effect of LCL-805 on ceramide levels. Sphingolipid profiling of MM-6 (A,B) and OCI-AML2 (C,D) cells treated with vehicle (DMSO) or LCL-805 (15 μM) for the indicated times. Levels of ceramide were evaluated by liquid chromatography mass spectrometry. Bars represent the average of four technical replicates from a representative experiment. Error bars represent +/- standard deviation. Statistical analyses represent Welch's ANOVA with Dunnett's T3 multiple comparisons test. * $p < 0.05$, ** $p < 0.005$, *** $p < 0.001$, **** $p < 0.0001$, ns = non-significant. D = DMSO; L = LCL-805. C## = fatty acid chain length. Total = sum of sphingolipid species.

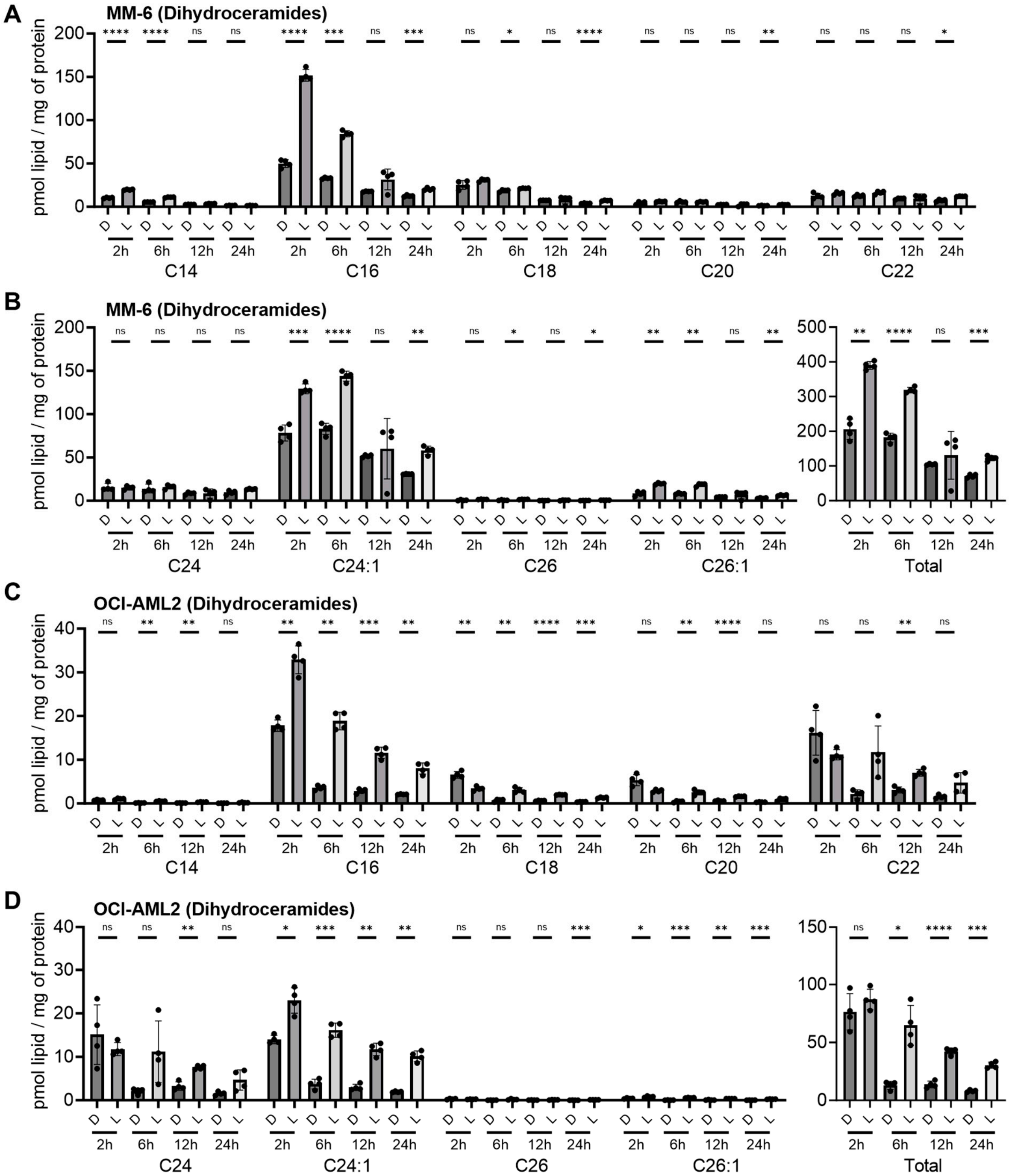

Figure S4. Effect of LCL-805 on dihydroceramide levels. Sphingolipid profiling of MM-6 (A,B) and OCI-AML2 (C,D) cells treated with vehicle (DMSO) or LCL-805 (15 µM) for the indicated times. Levels of dihydroceramide were evaluated by liquid chromatography mass spectrometry. Bars represent the average of four technical replicates from a representative experiment. Error bars represent +/- standard deviation. Statistical analyses represent Welch's ANOVA with Dunnett's T3 multiple comparisons test. * $p < 0.05$, ** $p < 0.005$, *** $p < 0.001$, **** $p < 0.0001$, ns = non-significant. D = DMSO; L = LCL-805. C## = fatty acid chain length. Total = sum of sphingolipid species.

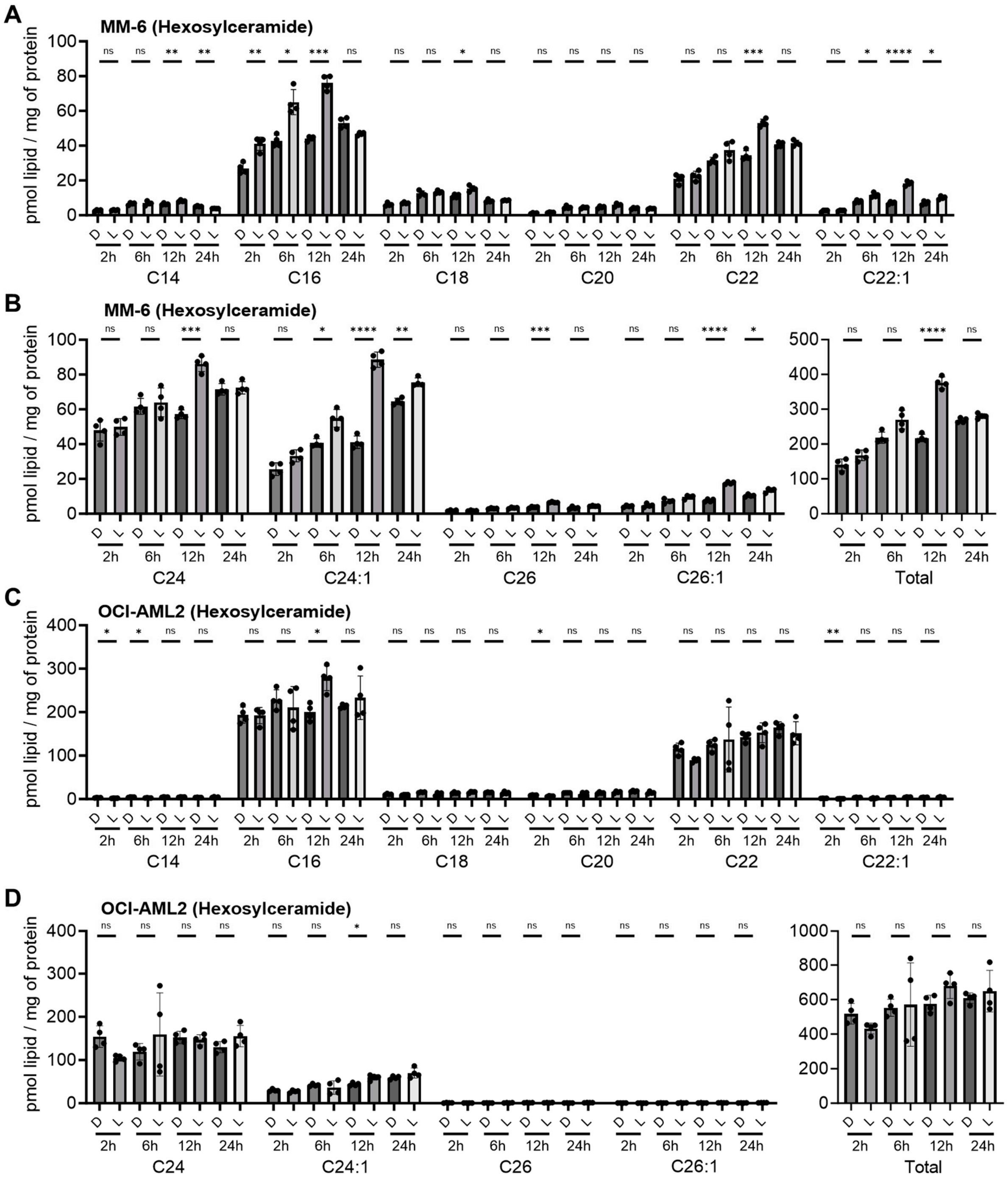

Figure S5. Effect of LCL-805 on hexosylceramide levels. Sphingolipid profiling of MM-6 (A,B) and OCI-AML2 (C,D) cells treated with vehicle (DMSO) or LCL-805 (15 μM) for the indicated times. Levels of hexosylceramide were evaluated by liquid chromatography mass spectrometry. Bars represent the average of four technical replicates from a representative experiment. Error bars represent +/- standard deviation. Statistical analyses represent Welch's ANOVA with Dunnett's T3 multiple comparisons test. * $p < 0.05$, ** $p < 0.005$, *** $p < 0.001$, **** $p < 0.0001$, ns = non-significant. D = DMSO; L = LCL-805. C## = fatty acid chain length. Total = sum of sphingolipid species.

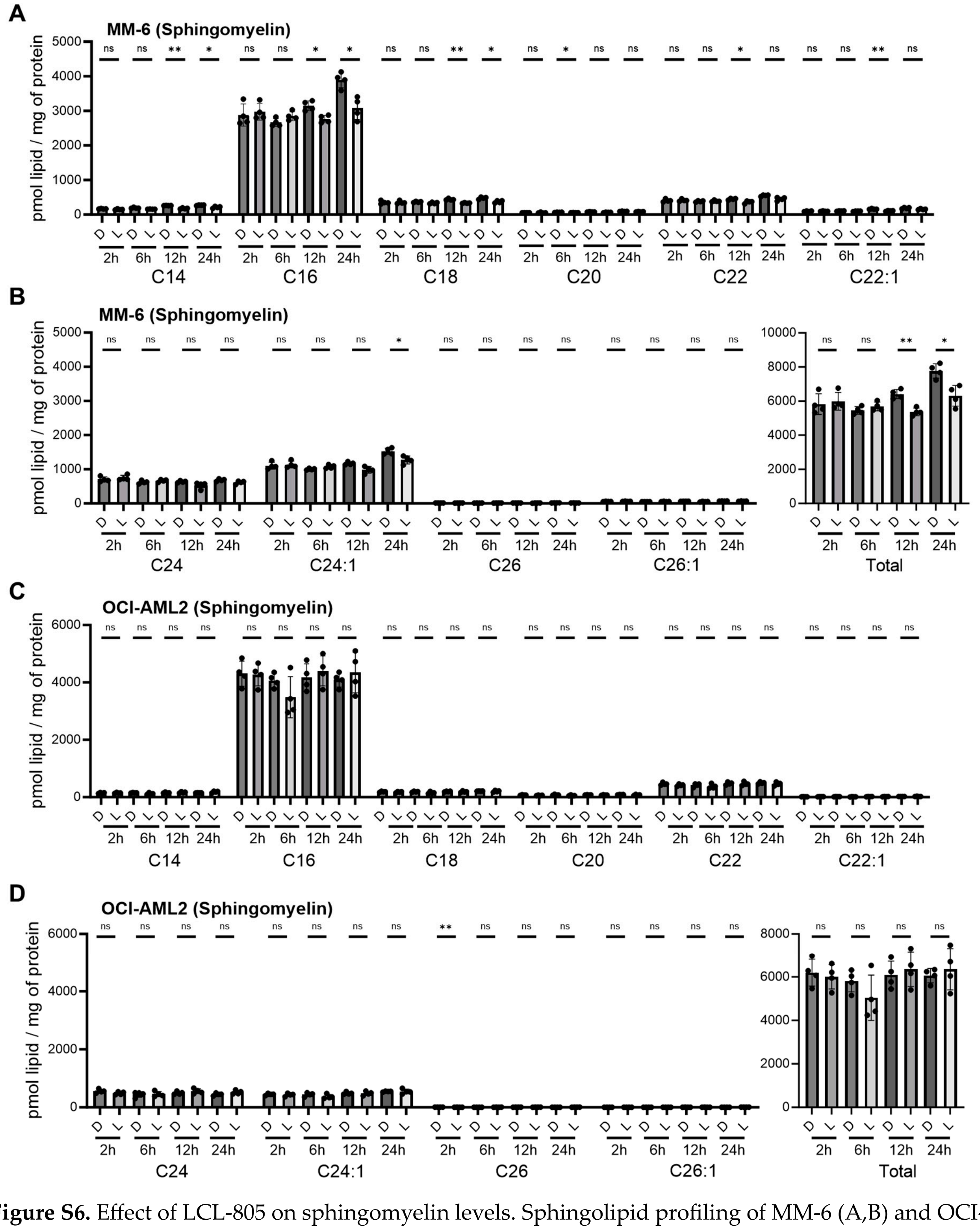

Figure S6. Effect of LCL-805 on sphingomyelin levels. Sphingolipid profiling of MM-6 (A,B) and OCI-AML2 (C,D) cells treated with vehicle (DMSO) or LCL-805 (15 μM) for the indicated times. Levels of sphingomyelin were evaluated by liquid chromatography mass spectrometry. Bars represent the average of four technical replicates from a representative experiment. Error bars represent +/- standard deviation. Statistical analyses represent Welch's ANOVA with Dunnett's T3 multiple comparisons test. * $p < 0.05$, ** $p < 0.005$, ns = non-significant. D = DMSO; L = LCL-805. C## = fatty acid chain length. Total = sum of sphingolipid species.

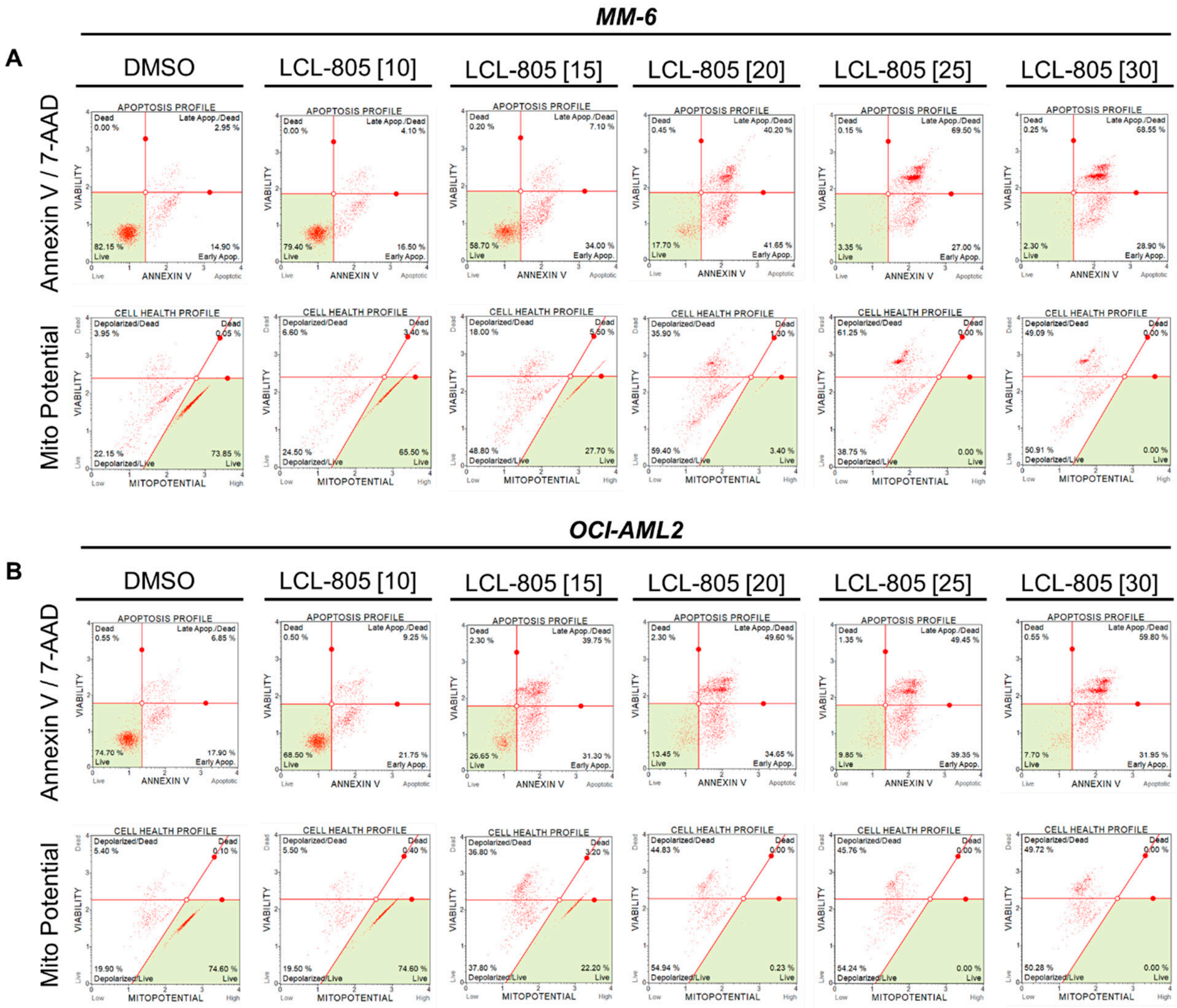

Figure S7. Flow cytometry-based detection of phosphatidylserine externalization and mitochondrial depolarization with LCL-805 treatment. MM-6 (A) and OCI-AML2 (B) were treated for 24 hours with vehicle (DMSO) or the indicated concentrations of LCL-805 (in µM, shown in brackets). Representative flow cytometry panels for the annexin V/7-AAD and mitochondrial depolarization assays are depicted. Data presented are representative experiments with at least three technical replicates, which were used to create Figure 4.

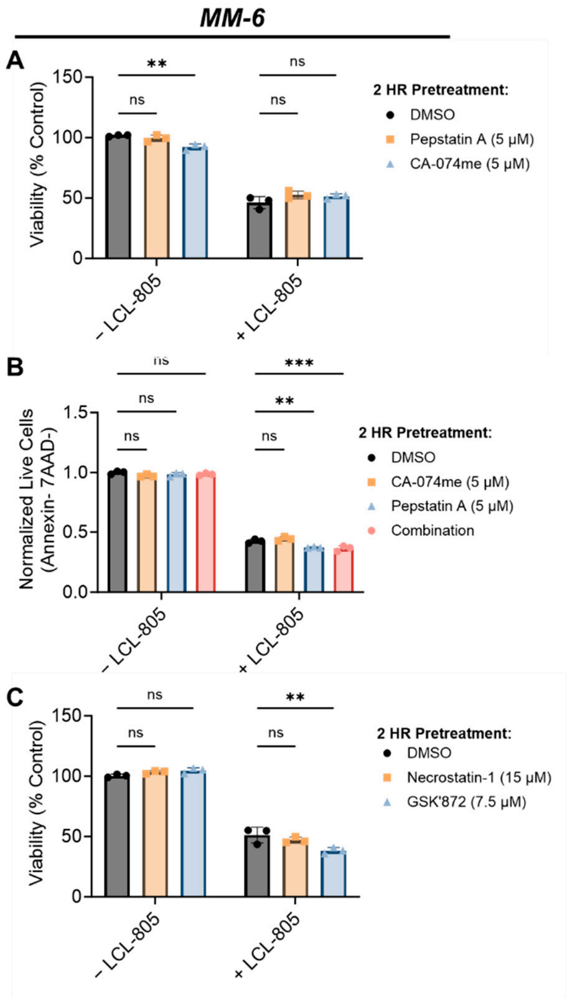

Figure S8. Inhibiting lysosomal cathepsins or necroptosis did not rescue LCL-805-mediated cytotoxicity. (A) Cell viability was determined by MTS assay of MM-6 cells pretreated with vehicle (DMSO), or lysosomal cathepsin inhibitors pepstatin A (5 μM), or CA-074me (5 μM) for 2 hours and subsequently treated with DMSO or LCL-805 (15 μM) for 24 hours. (B) Normalized live cell number by annexin and 7AAD flow cytometry of MM-6 cells pretreated with vehicle (DMSO), pepstatin A (5 μM), CA-074me (5 μM), or the combination for 2 hours and subsequently treated with DMSO or LCL-805 (15 μM) for 6 hours. (C) Cell viability was determined by MTS assay of MM-6 cells pretreated with vehicle (DMSO), or necroptosis inhibitors necrostatin-1 (15 μM), or GSK'872 (7.5 μM) for 2 hours and subsequently treated with DMSO or LCL-805 (15 μM) for 24 hours. Bars represent the average of three technical replicates from a representative experiment. Error bars represent +/- standard deviation. Statistical analyses represent two-way ANOVA with Tukey's multiple comparisons test. ** $p < 0.005$, *** $p < 0.001$, ns = non-significant.

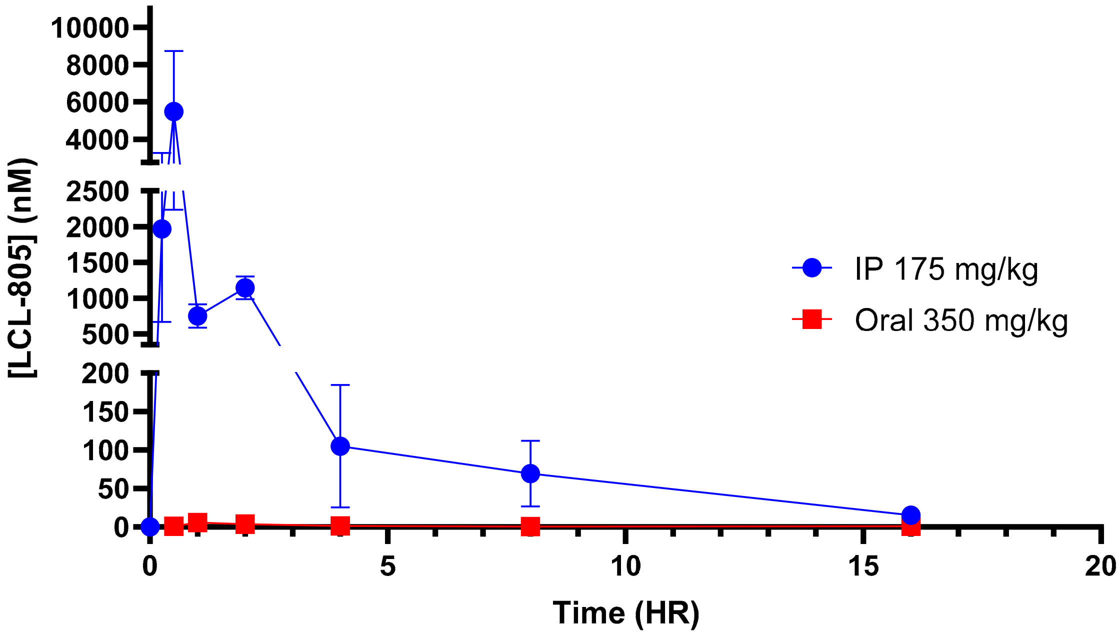

Figure S9. LCL-805 pharmacokinetics. B6(Cg)-Tyrc−2J/J (Albino B6) mice (The Jackson Laboratory, Bar Harbor, ME, USA) were given a single treatment of LCL-805 dissolved in saline at 300 mg/kg orally or 175 mg/kg intraperitoneally (n=3-5/group). Blood was collected via cardiac puncture at the indicated time points and LCL-805 levels were quantified via mass spectrometry. LCL-805 was most effectively delivered intraperitoneally due to rapid clearance following oral delivery and tail vein necrosis following intravenous injections. In a pilot study to test LCL-805 tolerability upon repeat dosing, 5–7-week-old male NOD.Cg-Rag1tm1Mom Il2rgtm1Wjl Tg (CMV-IL3,CSF2,KITLG)1Eav/J (NRG-S) mice (The Jackson Laboratory, Bar Harbor, ME, USA) exhibited severe toxicity and mortality with 2-3 intraperitoneal treatments of 90 mg/kg/day and 175 mg/kg/day. However, an intraperitoneal LCL-805 treatment with 40 mg/kg was well-tolerated by NRG-S mice treated once a day for five days (M-F) followed by a two-day break for two weeks. Therefore, LCL-805 was administered at 40 mg/kg intraperitoneally for in vivo efficacy studies using AML cell line-derived xenograft (CDX) mouse models. All animal experiments were conducted at the Penn State University College of Medicine under protocols approved by the Institutional Animal Care and Use Committee at Penn State, Hershey, PA, USA (IACUC #PROTO201246746). Error bars represent +/− standard deviation.

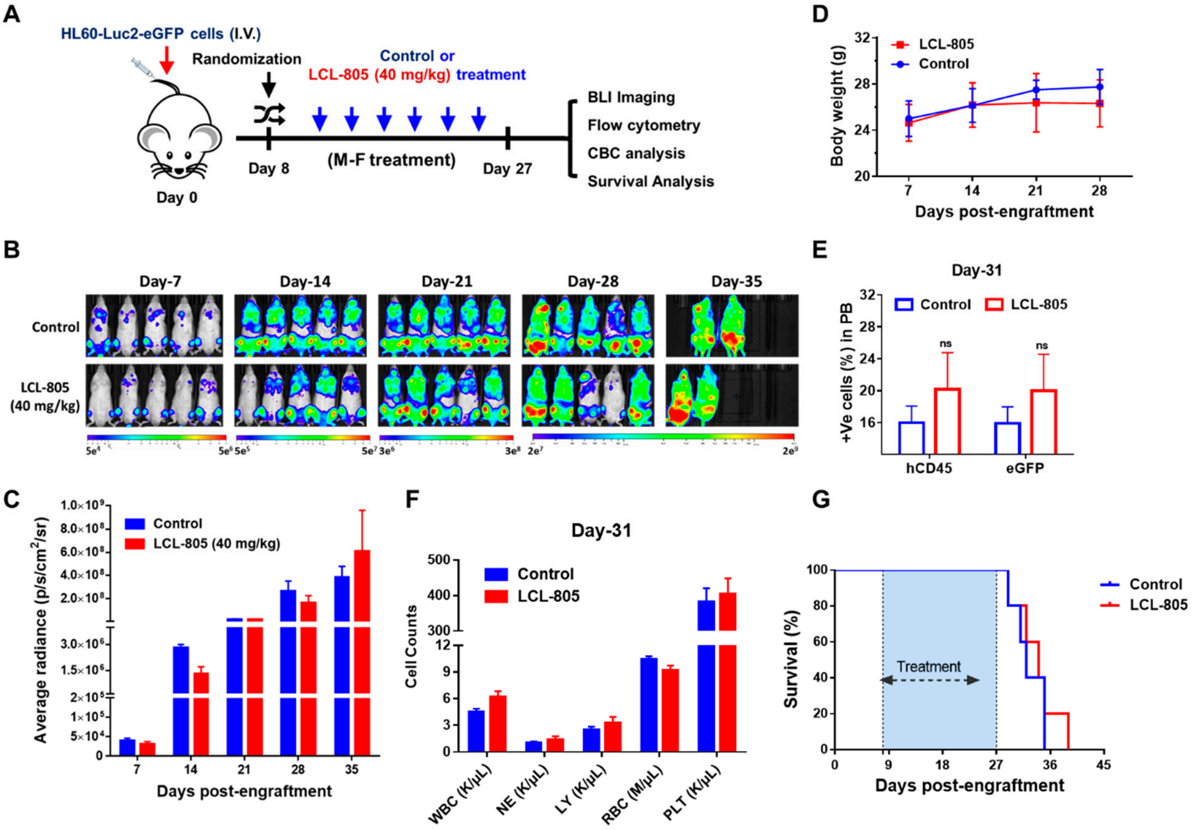

Figure S10. Lack of LCL-805 efficacy in an AML cell line-derived xenograft (CDX) mouse model. (A) 2×10^6 HL-60-Luc2-EGFP cells (HL-60 cells transduced with pFU-LUC2-eGFP lentivector and sorted for EGFP; plasmid gifted by Drs. Sanjiv Sam Gambhir and Tzuhua Dennis Lin) were injected intravenously into 5–7-week-old male NOD.Cg-Rag1tm1Mom Il2rgtm1Wjl Tg (CMV-IL3,CSF2,KITLG)1Eav/J (NRG-S) mice (The Jackson Laboratory, Bar Harbor, ME, USA) and randomized into control or LCL-805 groups (n=5) based on bioluminescence imaging (BLI) signals. LCL-805 dissolved in saline was administered intraperitoneally (40 mg/kg) once a day for five days (M-F) followed by a two-day break for 3 weeks. (B) Bioluminescence images of control and LCL-805-treated CDX mice on the indicated days. Animals were imaged using the IVIS Lumina LT Series III imaging system (Perkin Elmer, Waltham, MA, USA) 7 min after D-luciferin (Gold Biotechnology, St. Louis, MO, USA) injection (150 mg/kg; IP). (C) Quantified BLI signals measured over time. Photons emitted were quantified, analyzed, and plotted as average radiance (photons/s/cm2/steradian), using the Living Image software 4.8 (Perkin Elmer, Waltham, MA, USA). (D) Weights (g) of the control and LCL-805-treated mice over time. Values are represented as the mean +/− standard deviation. (E) Leukemic burden assessed from peripheral blood on day 31 post-engraftment by flow cytometry staining for human CD45 and GFP markers (GFP expression (FITC), anti-human CD45-PE (BD Biosciences, Franklin Lakes, NJ, USA, #555483)), anti-mouse CD45-BV650 (BD Biosciences, #563410), and dead cell exclusion dye, 7-AAD (BioLegend, San Diego, CA, USA, #420404). Data were acquired using the BD LSRFortessa Flow Cytometer (BD Biosciences) at the Core Facility of Pennsylvania State University College of Medicine (RRID:SCR_021134) and processed using FlowJo v10.0 (BD Biosciences). (F) Complete blood count analysis from CDX mouse peripheral blood on day 31 post-engraftment of leukocytes (WBC = white blood cell; NE = neutrophil; LY = lymphocyte), red blood cells (RBC), and platelets (PLT) using the Hemavet 950 analyzer (Drew Scientific, Miami Lakes, FL, USA). (G) Kaplan–

Meier survival analysis of the LCL-805-treated group versus the control group. All animal experiments were conducted at the Penn State University College of Medicine under protocols approved by the Institutional Animal Care and Use Committee at Penn State, Hershey, PA, USA (IACUC #PROTO201246746). Data are the mean +/− standard error of the mean. Statistical analyses represent two-way ANOVA. ns = non-significant.

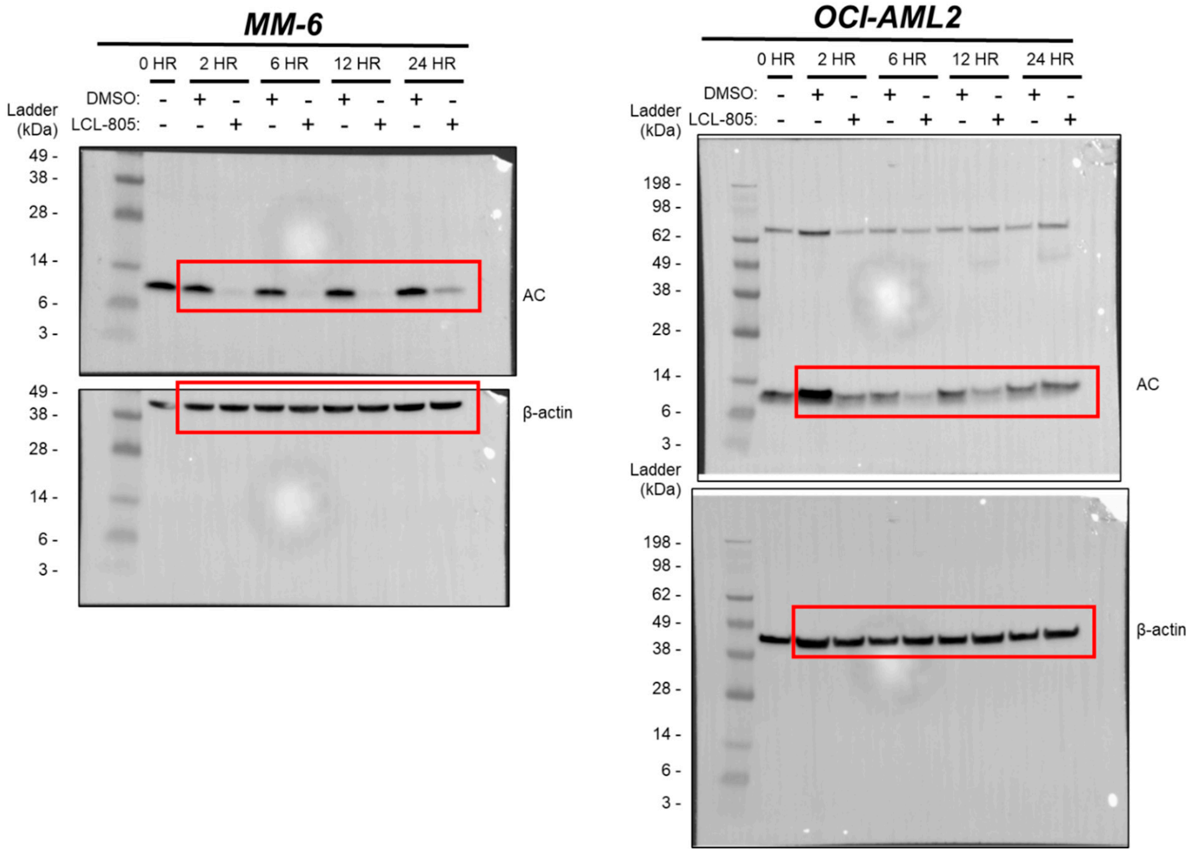

Figure S11. Full, annotated, unedited western blots for Figure 1C. Red boxes were cropped for the figure.

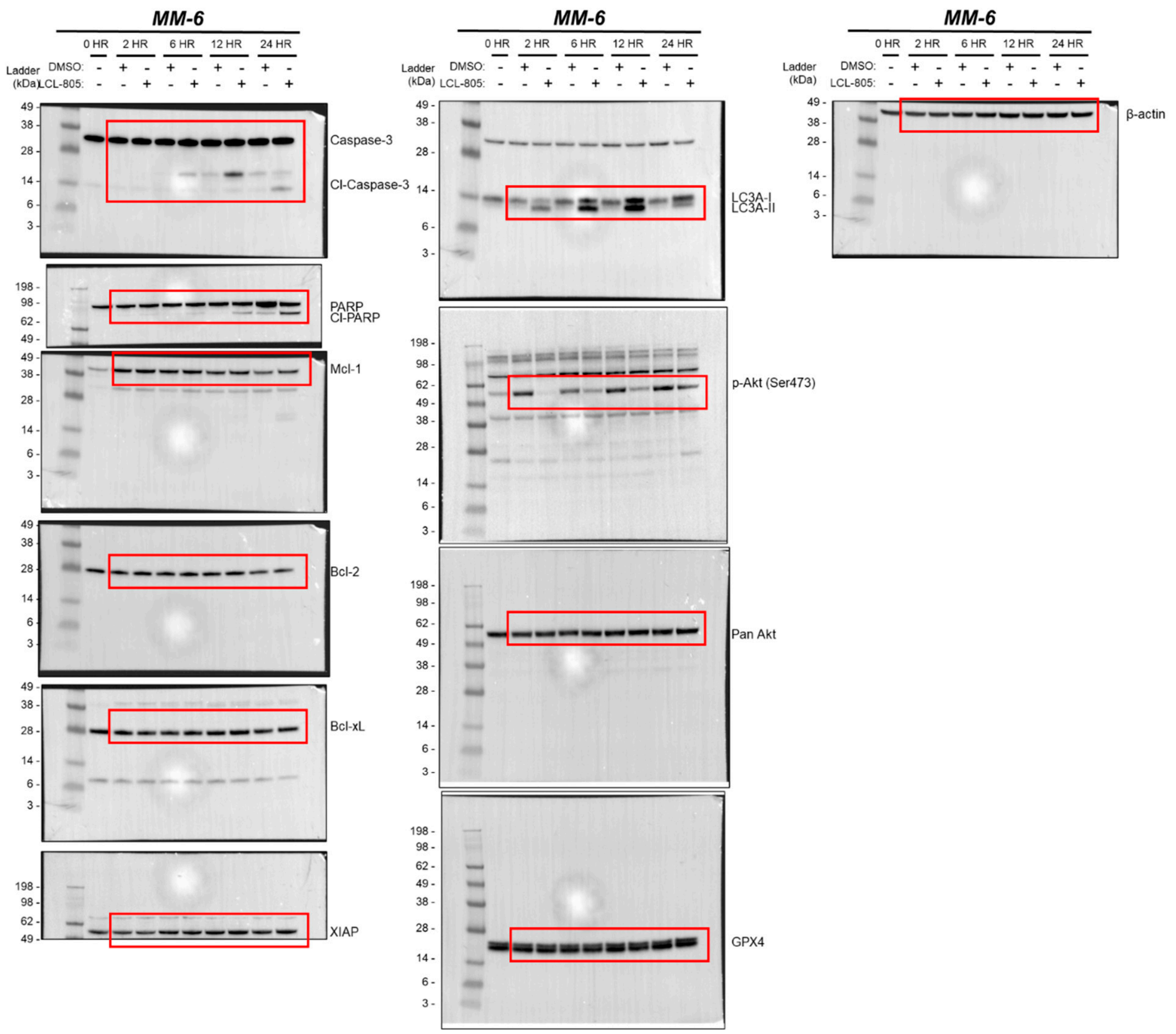

Figure S12. Full, annotated, unedited western blots for Figure 5A. Red boxes were cropped for the figure.

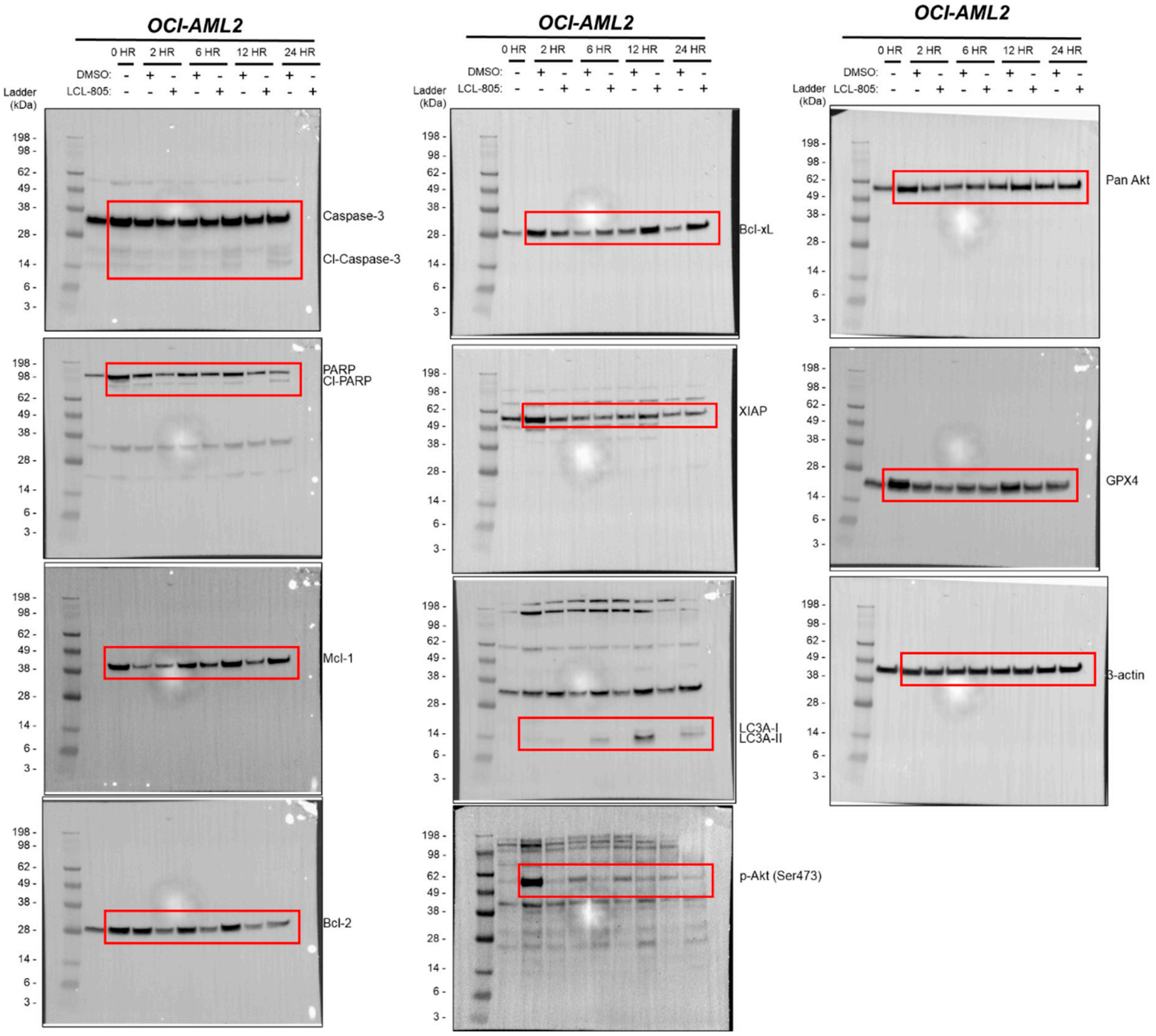

Figure S13. Full, annotated, unedited western blots for Figure 5B. Red boxes were cropped for the figure.

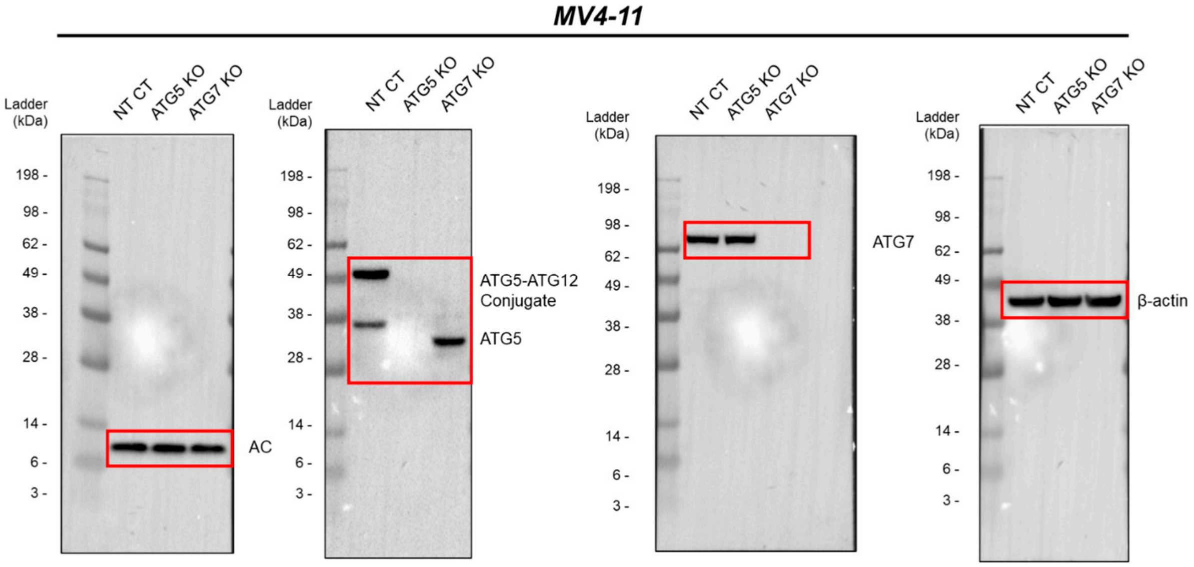

Figure S14. Full, annotated, unedited western blots for Figure 6G. Red boxes were cropped for the figure.

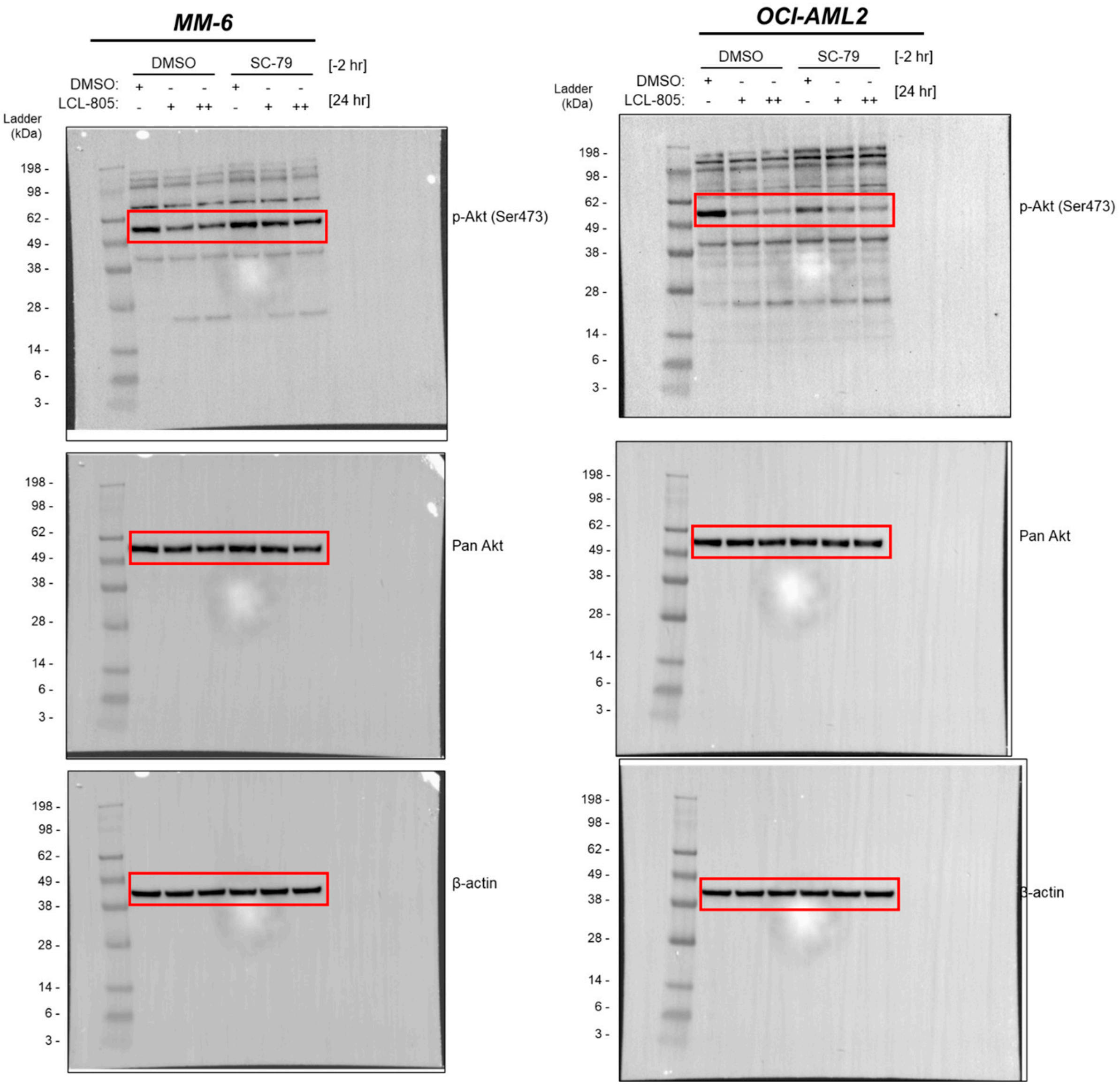

Figure S15. Full, annotated, unedited western blots for Figure 7C. Red boxes were cropped for the figure.

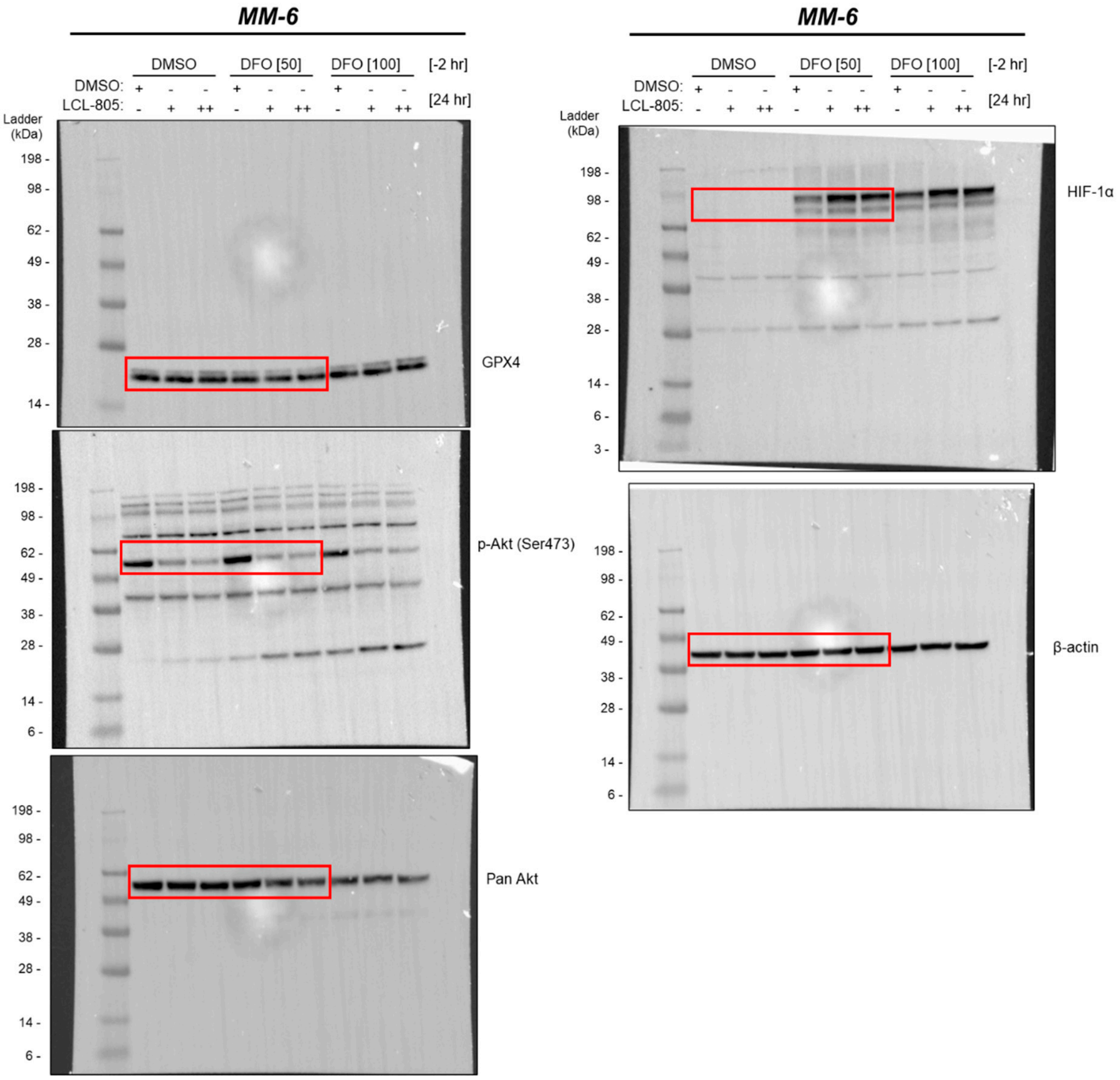

Figure S16. Full, annotated, unedited western blots for Figure 8G (MM-6). Red boxes were cropped for the figure.

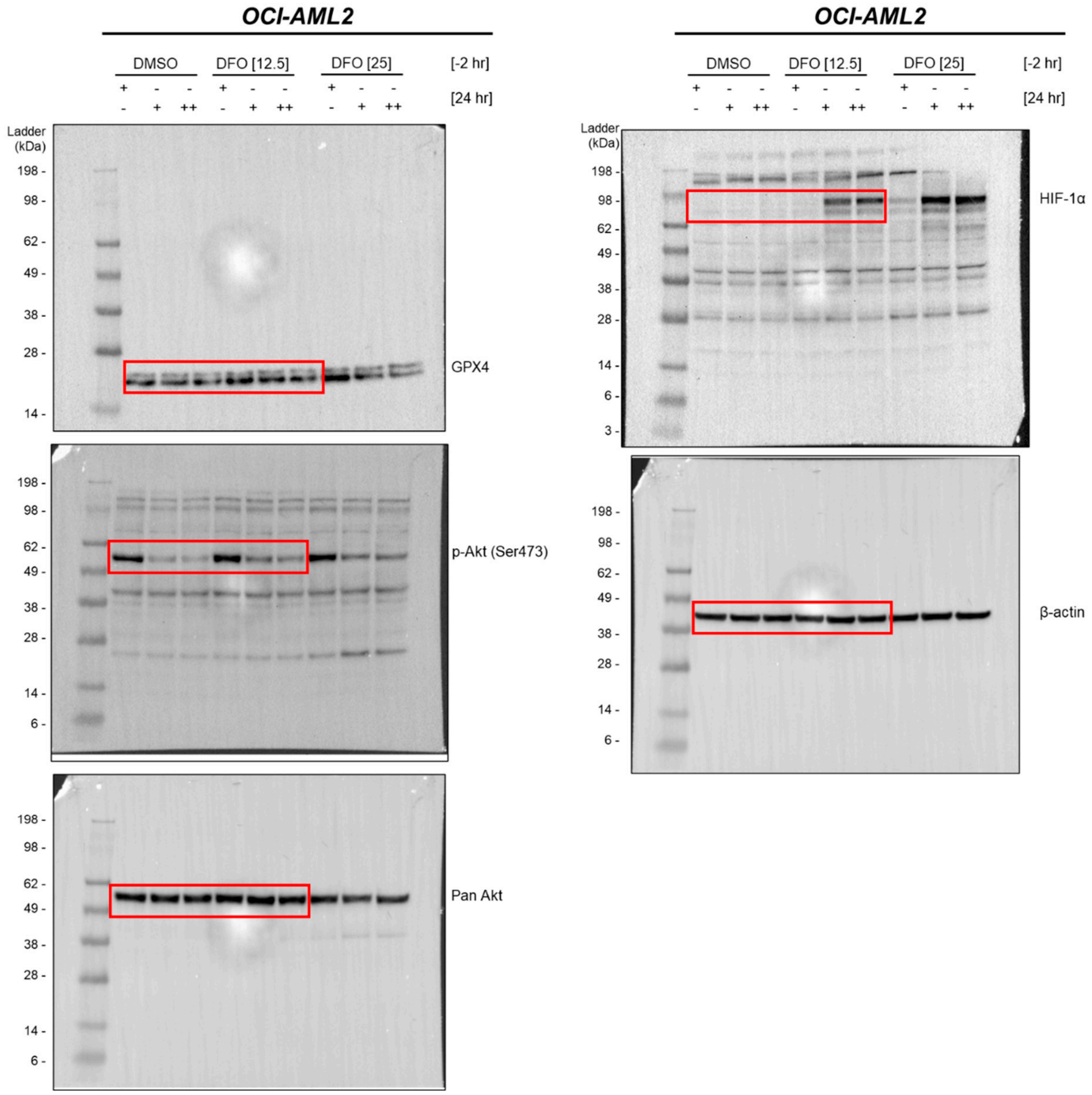

Figure S17. Full, annotated, unedited western blots for Figure 8G (OCI-AML2). Red boxes were cropped for the figure.

Table S1. AML patient sample cohort.

ID	Age	Gender	Disease Stage	Sample Type	Blast %	Complete Cytogenetics	LCL-805 EC50 (μM)	Notes
134	60.5	M	Dx	PBMC	5.0	*Not Given*	15.8	
150	52.0	M	Dx	PBMC	85.0	46,XY	*Not Tested*	
215	74.6	M	Dx	PBMC	94.0	44,XY,add(9)(p13),-16,-17,+mar	14.4	

218	35.9	F	Dx	PBMC	94.0	46,XX	*Not Reached*	
245	48.0	F	Dx	PBMC	73.0	47,XX,+9	10.6	
329	71.6	F	Dx	PBMC	75.0	45,XX,inv(3)(q21q26.2),-7	18.7	
591	52.7	M	Rel>1	PBMC	*Not Reported*	46,XY, del(5)(q15q33)	*Not Reached*	
600	74.3	F	Rel 1	PBMC	*Not Reported*	46,XX	*Not Reached*	
601	70.5	F	Dx	PBMC	87.0	46,XY,ider(11)(q10)inv(11)(q13q25)	18.0	
665	27.0	M	Dx	PBMC	58.0	46,XY,inv(16)(p13.1q22)	10.9	665/667 derived from same patient; both PBMC samples colleected at different dates
667	27.0	M	Dx	PBMC	19.8	46,XY,inv(16)(p13.1q22)	15.2	665/667 derived from same patient; both PBMC samples colleected at different dates
730	62.2	M	Dx	PBMC	25.5	46,XY	15.8	
739	58.5	M	Dx	PBMC	2.6	46,XY	17.9	
765	63.5	M	Dx	PBMC	77.0	47,XY,t(16;16)(p13.1;q22),+22	*Not Reached*	
767	38.6	F	Rel>1	PBMC	24.0	46,XX	*Not Reached*	
772	55.1	F	Dx	PBMC	57.7	46,XX	*Not Reached*	
796	63.3	M	Dx	PBMC	85.0	45-47,X,-Y,der(1)t(1;3)(p36.1;p21),+7,r(16),-19,+20	13.4	
801	47.5	M	Dx	PBMC	22.4	46,XY	*Not Reached*	801/802 derived from same patient; 801 = PBMC; 802 = marrow
802	47.5	M	Dx	Marrow	43.0	46,XY	*Not Reached*	801/802 derived from same patient; 801 = PBMC; 802 = marrow
807	28.7	F	Receiving tx	PBMC	67.3	46,XX	*Not Reached*	
827	74.4	M	Dx	PBMC	93.0	46,XY	16.2	
841	76.7	F	Dx	PBMC	80.5	46,XX	*Not Reached*	
842	59.9	M	Dx	PBMC	87.0	46,XY	18.5	
850	60.3	F	Dx	PBMC	53.0	46,XX	*Not Reached*	
884	47.5	M	Rel 1	PBMC	77.0	46,XY	17.5	
933	58.2	F	Dx	PBMC	90.8	46,XX,t(8;21)(q22;q22),t(10;13)(q22;q12)	*Not Reached*	
934	71.6	F	Dx	PBMC	98.0	46,XX	*Not Reached*	
936	55.2	M	Dx	PBMC	37.7	44,XY,del(1)(q42),-7,add(8)(p23),-12	*Not Reached*	
939	44.8	F	Dx	PBMC	93.8	46,XX	18.6	
954	22.4	M	Dx	PBMC	68.0	46,XY	16.6	
967	41.8	F	Dx	PBMC	51.9	46,XX	15.8	

976	64.1	F	Dx	PBMC	65.4	46,XX	*Not Reached*	
983	74.2	F	Dx	PBMC	73.0	46,XX,add(9)(q13),hsr(9)(q13)	15.7	
990	66.1	M	Dx	PBMC	61.5	46,XY,del(13)(q12q14)	*Not Reached*	
996	60.2	M	Dx	PBMC	23.9	46,XY,inv(16)	14.9	
997	66.1	M	Dx	PBMC	87.7	47,XY,+21	15.0	
1006	47.9	M	Dx	PBMC	73.8	46,XY,add(5)(q31)	*Not Reached*	
1008	72.5	M	Dx	PBMC	35.2	47,XY,+8,add(10)(p11.2),der(22)t(1;22)(q11;p13)	*Not Reached*	
1017	52.5	M	Dx	PBMC	9.0	46,XY,t(3;21)(q26.2;q22)	13.7	
1020	58.9	M	Dx	Marrow	48.5	46,XY,t(6;9)(p23;q34),t(8;13;11)(q22;q12;q23)	18.5	1020/1021 derived from same patient; 1020 = marrow; 1021 = PBMC
1021	58.9	M	Dx	PBMC	79.9	46,XY,t(6;9)(p23;q34),t(8;13;11)(q22;q12;q23)	*Not Reached*	1020/1021 derived from same patient; 1020 = marrow; 1021 = PBMC
1033	57.9	F	Dx	PBMC	24.3	46,XX,del(6)(q21),add(17)(p13)	16.6	
1035	67.7	M	Dx	PBMC	37.0	47,XY, +mar, Inv(3)(q21q26.2),-7	*Not Reached*	
1044	70.1	M	Dx	PBMC	63.8	46,XY,i(17)(q10)	*Not Reached*	
1048	61.1	F	Dx	PBMC	24.5	46,XX	12.7	
1065	65.2	F	Dx	PBMC	35.0	46,XX,t(9;22)(q34;q11.2)	11.6	
1071	80.7	M	Dx	PBMC	98.0	47,XY,+8	10.5	1071/1072 derived from same patient; 1071 = PBMC; 1072 = marrow
1072	80.7	M	Dx	Marrow	73.0	47,XY,+8	19.1	1071/1072 derived from same patient; 1071 = PBMC; 1072 = marrow
1080	55.8	M	Dx	Marrow	74.5	46,XY	14.6	1080/1082 derived from same patient; 1080 = marrow; 1082 = PBMC
1081	58.4	M	Dx	PBMC	95.0	46,XY	17.7	
1082	55.8	M	Dx	PBMC	9.4	46,XY	*Not Reached*	1080/1082 derived from same patient; 1080 = marrow; 1082 = PBMC
1099	83.7	M	Dx	PBMC	61.0	46,XY	*Not Reached*	
1103	41.3	F	Dx	PBMC	86.0	46,XX	14.4	
1321	40.3	F	Dx	PBMC	81.2	46,XX	*Not Reached*	
1356	64.4	M	Dx	PBMC	72.5	46,XY,inv(16)(p13.1q22)	18.7	
1361	58.8	F	Dx	PBMC	90.0	46,XX	*Not Reached*	
1374	36.5	M	Dx	PBMC	86.7	46,XY,t(9;22)(q34;q11.2)	*Not Reached*	
1380	29.9	M	Dx	PBMC	60.0	46,XY	12.7	
PBL 10005	75.0	M	Dx	PBMC	89.5	46,XY	19.8	

PBL 10016	71.0	M	Dx	PBMC	*Not Reported*	46,XY	16.8
PBL 10173	64.0	M	Dx	PBMC	52.3	46,XY	19.3
PBL 10215	72.0	F	Dx	PBMC	14.0	46,XX	*Not Reached*
PBL 10261	66.0	F	Dx	PBMC	12.5	46,XX	*Not Reached*
PBL 10278	59.0	M	Dx	PBMC	20.9	47,XY,+8,del(11)(q14q23),der(21)t(1;21)(q21;p11.2)	*Not Reached*
PBL 8231	56.0	F	Dx	PBMC	54.4	46,XX,der(11)del(11)(q23q23)t(11;17)(q23;q?25)	14.4
PBL 8426	30.0	F	Dx	PBMC	84.0	46,XX,t(11;19)(q23;p13.1)	*Not Reached*
PBL 8870	68.0	M	Dx	PBMC	31.2	46,XY	*Not Reached*
PBL 8934	67.0	M	Dx	PBMC	93.5	47,XY,+8,inv(16)(p13q22)	*Not Reached*
PBL 9097	50.0	M	Dx	PBMC	26.0	46,XY,inv(16)(p13.1q22)	19.3
PBL 9542	66.0	M	Dx	PBMC	56.0	45,XY,-7	*Not Reached*
PBL 9785	61.0	M	Dx	PBMC	69.0	46,XY	18.4
PBL 9948	61.0	M	Dx	PBMC	53.2	47,XY,+8,del(5)(q31q35)	*Not Reached*
PBL 9958	54.0	M	Dx	PBMC	69.0	48,XY,+9,+13	*Not Tested*
